# Evaluation of the Effectiveness of Semolina in Improving the Bond Strength of Metal Alloys and Acrylic Resins for the Fabrication of Hybrid Prostheses for Implant Restoration: An In Vitro Study

**DOI:** 10.7759/cureus.65686

**Published:** 2024-07-29

**Authors:** Urvi R Echhpal, Khushali K Shah, Subhasree R

**Affiliations:** 1 Prosthodontics and Implantology, Saveetha Dental College and Hospitals, Saveetha Institute of Medical and Technical Sciences, Saveetha University, Chennai, IND

**Keywords:** dmls, metal casting, cobalt chromium, hybrid prosthesis, semolina

## Abstract

Introduction

Fixed prosthodontic solutions for restorations of multiple implants are often done using acrylic-metal hybrid prosthesis. These restorations have a high failure rate due to poor bond strength between metal and acrylic, despite using digital attachments and primers. This study has been done to demonstrate an economical yet effective method of increasing shear bond strength between acrylic and metal.

Materials and methods

Disc specimens of 10 mm diameter and 2 mm thickness were designed using the Meshmixer version 3.5 software (Autodesk Inc., San Rafael, USA). The standard tessellation (STL) file was imported and sent for direct metal laser sintering (DMLS) in cobalt-chromium (CoCr), titanium (Ti), and wax milling. The groups were defined. Ten samples were included in each group. Group 1: Conventional Casting in CoCr; Group 2: Conventional Casting with Opaquer (CoCr); Group 3: DMLS CoCr; Group 4: DMLS CoCr with Opaquer; Group 5: DMLS Ti; Group 6: DMLS Ti with Opaquer; Group 7: Casting with Semolina (CoCr); and Group 8: Casting with Semolina and Opaquer (CoCr). Wax-up was performed using double wax sheet thickness of modeling wax. Flasking and acrylization were done using the injection moulding technique. A universal testing machine was used for shear bond strength. Results were tabulated and statistical analysis was done using IBM SPSS Statistics for Windows (IBM Corp., Armonk, USA). The Shapiro-Wilk test was done to confirm the normality of the data, followed by the one-way analysis of variance (ANOVA) test to compare the shear bond strength between metal alloys and heat cure acrylic resin.

Results

The one-way ANOVA results reveal significant differences in bond strengths among the various groups. Conventional Casting with Opaquer showed no significant difference (p=0.335), but there were notable improvements in bond strength when compared to DMLS CoCr, DMLS CoCr with Opaquer, DMLS Ti, DMLS Ti with Opaquer, Semolina, and Semolina with Opaquer, with DMLS Ti having the largest mean difference (3.368). Conventional Casting with Opaquer significantly outperformed all groups, especially Semolina, which showed the highest mean difference (7.558). Similar trends were seen with DMLS CoCr and DMLS Ti, where Semolina consistently demonstrated superior bond strength. Overall, Semolina produced the highest bond strengths across all conditions. These results underscore the importance of material selection and treatment in enhancing bond strength.

Conclusion

Semolina can be used as an economical and reliable method to increase the shear bond strength between acrylic and metal bonding. Its ability to burn out during dewaxing without any remains or remnants in mould deems it a useful material for increasing mechanical bonding.

## Introduction

Fixed prosthetic rehabilitation of full mouth implants stands to be the most challenging aspect of implant dentistry for most clinicians. Dentistry is experiencing a revolution in digital practice. Digital dentistry has a steep learning curve, which every clinician experiences [1]. The choice of restorations is based on the availability of space, implant number and position, biting forces, and expectations of the patient. Often, the restoration of multiple implants is an acrylic hybrid prosthesis, which consists of a smaller metal framework over which an acrylic denture base and acrylic teeth are used [2]. They are commonly used due to their cost-effectiveness and ease of fabrication. However, they have been reported to have a large number of failures, owing to poor bonding of acrylic and metal, like debonding of acrylic teeth and debonding of denture base from metal bars. Often, compensation for low shear bond strength (SBS) between alloys of metal and acrylic may increase the thickness of the denture base; however, the over-contoured restoration affects the balance between the patient's cheek and tongue [3].

Dental laboratories and clinicians have tried various design modifications to increase the retention of acrylic on metal bars. Several designing software have incorporated a variety of bar cross-sections as well as attachment options to improve retention [4]. Direct metal laser sintering (DMLS) is increasingly preferred over conventional lost wax casting due to the higher likelihood of casting errors in the latter [5]. While DMLS offers high precision, it can limit clinicians and technicians because of the design options available within the software. DMLS is commonly used with metals like titanium (Ti) and cobalt chromium (CoCr) alloys. A hybrid technique that combines digital and conventional methods allows practitioners to achieve the precision of digital fabrication while maintaining the customizability of traditional approaches.

The only restrictions are the ones we create. This study aims to outline the benefits of a hybrid technique, allowing customization of manual techniques and the precision of fit of digital techniques. The objective of this study is to assess the alteration in shear bond strength between metal alloys and acrylic by incorporating semolina prior to investing. The null hypothesis was defined as stating that there would be no significant difference in the shear bond strength with and without the incorporation of semolina. By focusing on the metal-acrylic interface, this study seeks to address the common failures in hybrid prostheses and improve their overall durability and reliability.

## Materials and methods

Study setting 

**Figure 1:**
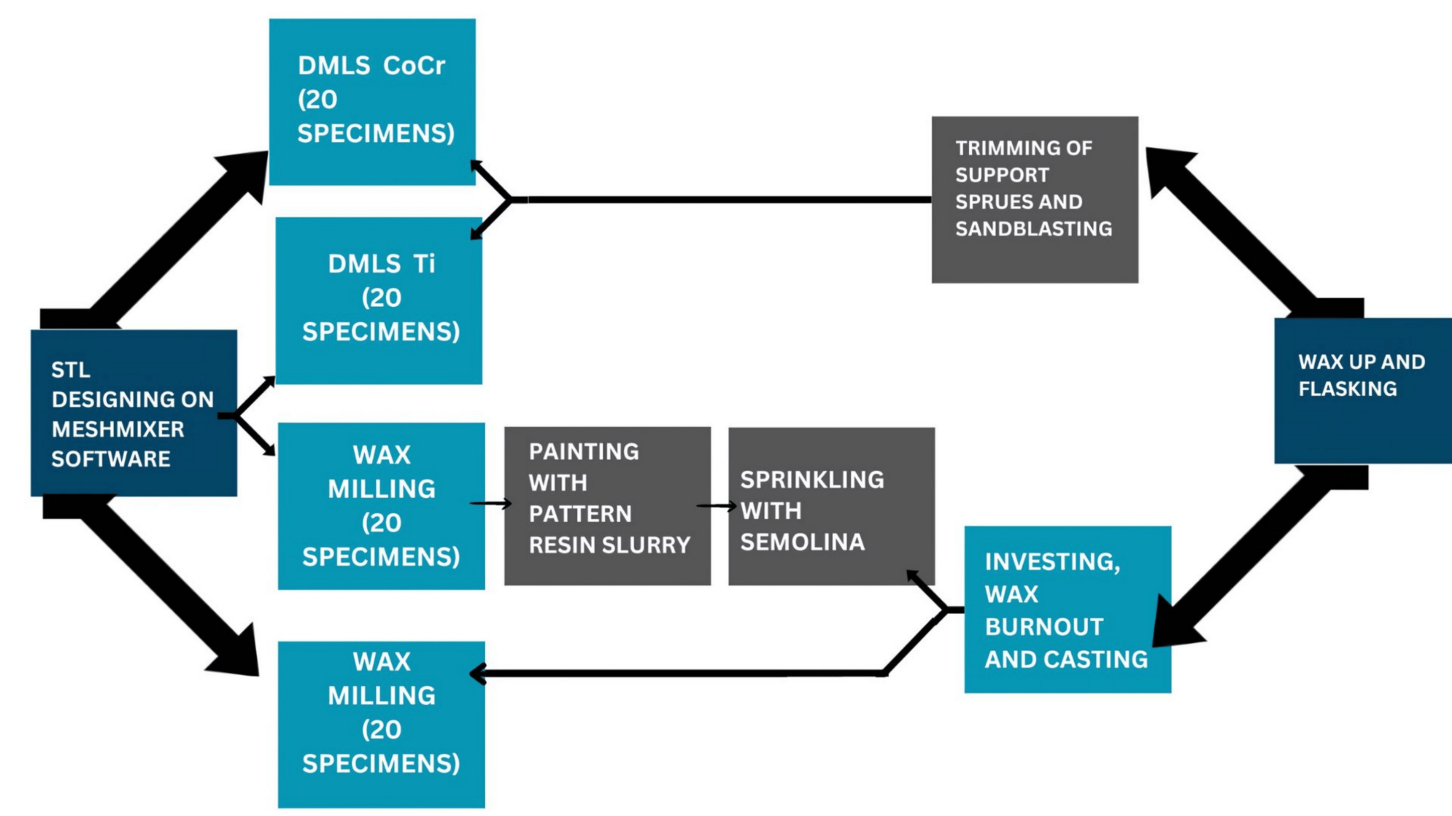
Workflow of the study DMLS: direct metal laser sintering; Ti: titanium; CoCr: cobalt-chromium

This in vitro study was done at a research facility in Poonamallee, Tamil Nadu, India. Approval for conducting this study was granted by the Scientific Review Board (SRB) of the institute under the approval number SRB/SDC/PROSTHO-2106/24/089. The workflow of the study was finalized as outlined in Figure 1.

Estimation of the sample size 

The G*Power software version 3.1.2 (Heinrich-Heine-Universität Düsseldorf, Düsseldorf, Germany) was utilized to compute the sample size for this investigation. Reference was made to the mean and standard deviation from a previous study by Külünk et al., with parameters set at p<0.50, a significance level of 5%, and a study power of 0.95. This calculation indicated that a sample size of 10 was necessary [6].

Obtaining test groups

Substructure Fabrication

**Figure 2:**
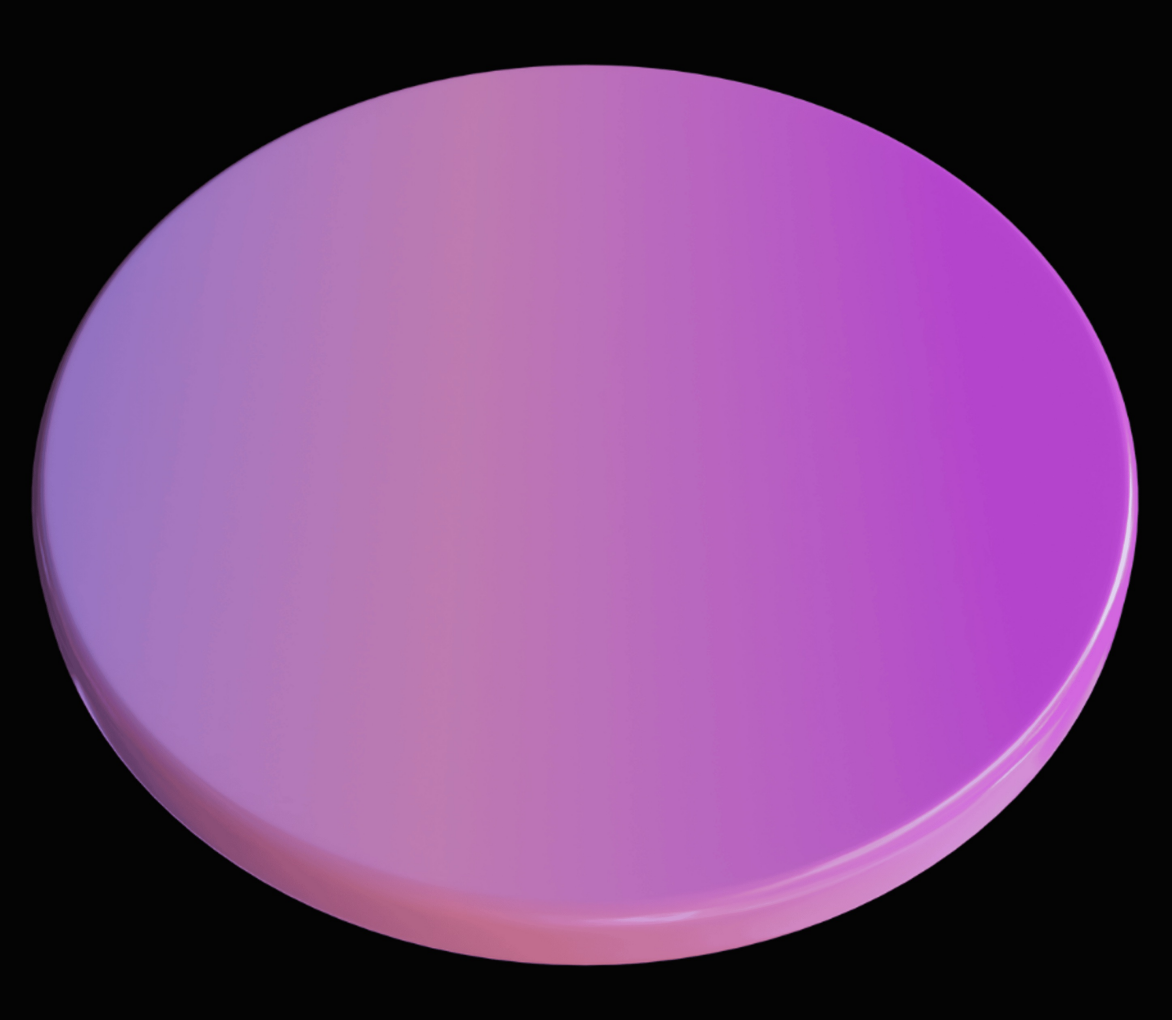
Standard tessellation language file of the specimen

The disc was designed using Meshmixer version 3.5 software (Autodesk Inc., San Rafael, USA) with a diameter of 10 mm and a thickness of 2 mm (Figure 2).

**Figure 3:**
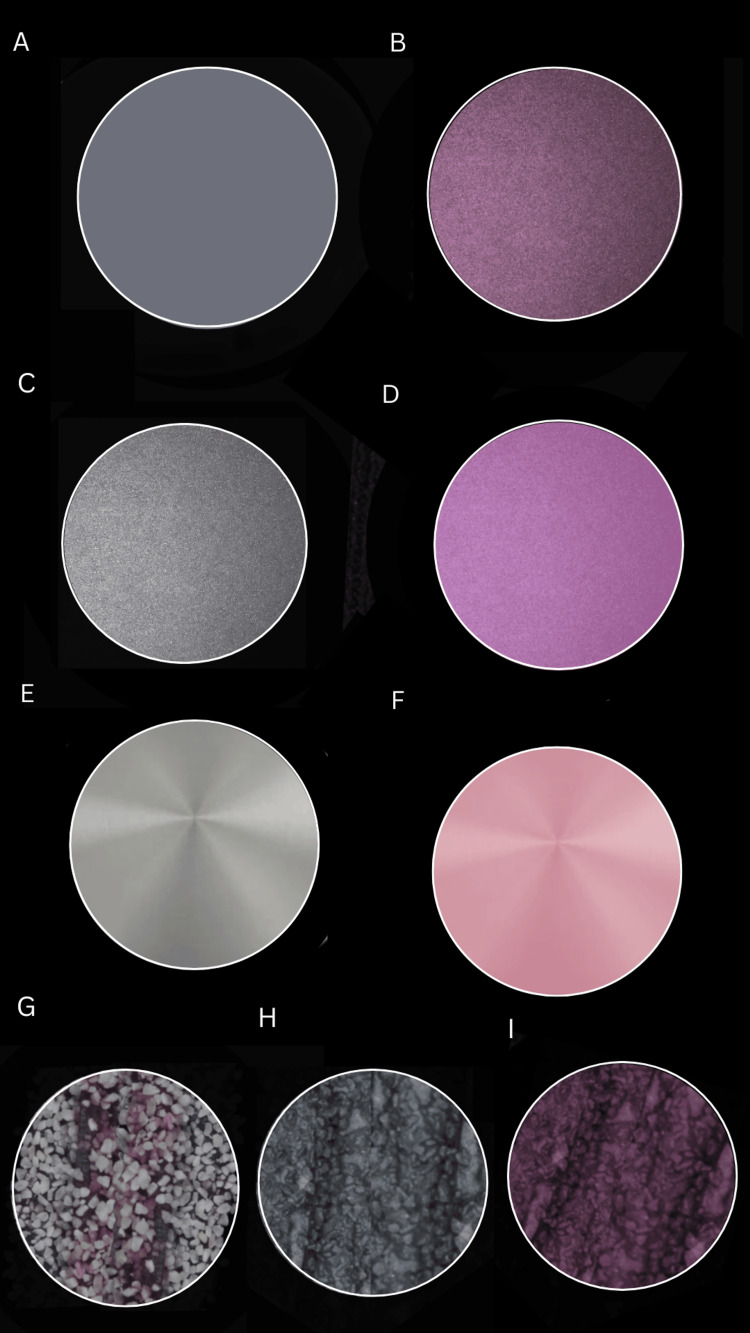
Specimens after fabrication (A) Conventionally casted specimen; (B) Conventionally casted specimen painted with opaquer; (C) Direct metal laser sintered specimen (CoCr); (D) Direct metal laser sintered specimen (CoCr) with opaquer; (E) Direct metal laser sintered specimen (Ti); (F) Direct metal laser sintered specimen (Ti) with opaquer; (G) Wax specimen painted with pattern resin and sprinkled with semolina; (H) Semolina specimen after casting; (I) Semolina specimen painted with opaquer Ti: titanium; CoCr: cobalt-chromium

The standard tessellation language (STL) file was exported. The STL was then used for wax milling using Upcera wax blank (Upcera, China) in a milling machine (Roland, Japan; Model DWX-4W). DMLS was used for printing the disc in CoCr and Ti EOS M100 (EOS, Krailling, Germany). A total of 10 wax samples were directly invested. The 10 wax samples were painted using pattern resin and then sprinkled with semolina purchased from a local grocery store (Figure 3).

The groups were defined, as shown in Table 1.

**Table 1:** Definition of groups DMLS: direct metal laser sintering; Ti: titanium; CoCr: cobalt-chromium

Group	Features
1	Conventional Casting (CoCr)
2	Conventional Casting with Opaquer (CoCr)
3	DMLS Cocr
4	DMLS CoCr with Opaquer
5	DMLS Titanium
6	DMLS Titanium with Opaquer
7	Casting with Semolina (CoCr)
8	Casting with Semolina and Opaquer (CoCr)

Wax-up and Acrylization

**Figure 4:**
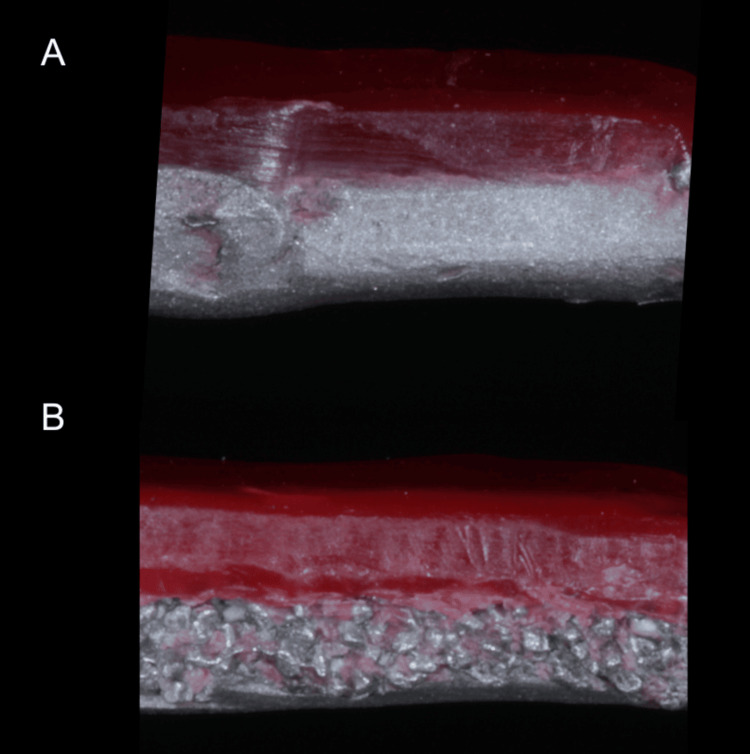
Lateral view of specimens after wax-up (A) Group 1 specimen; (B) Group 7 specimen Image showing the difference between the wax and metal interface after treatment with semolina. The remaining groups appeared similar to (A) and were therefore not included.

Wax-up of specimens was performed using modeling wax (Maarc Dental, India) obtained from a dental material store using double wax sheet thickness (Figure 4). Flasking was done using Elite Master (Zhermack, Italy). Dewaxing was carried out after which specimens of groups 2, 4, 6, and 8 were painted with gingival opaquer (GUM-O, Shofu, Japan), and injection moulding was carried out for acrylization using an Ivo base cartridge (Ivoclar Vivadent, USA).

Testing of SBS

**Figure 5:**
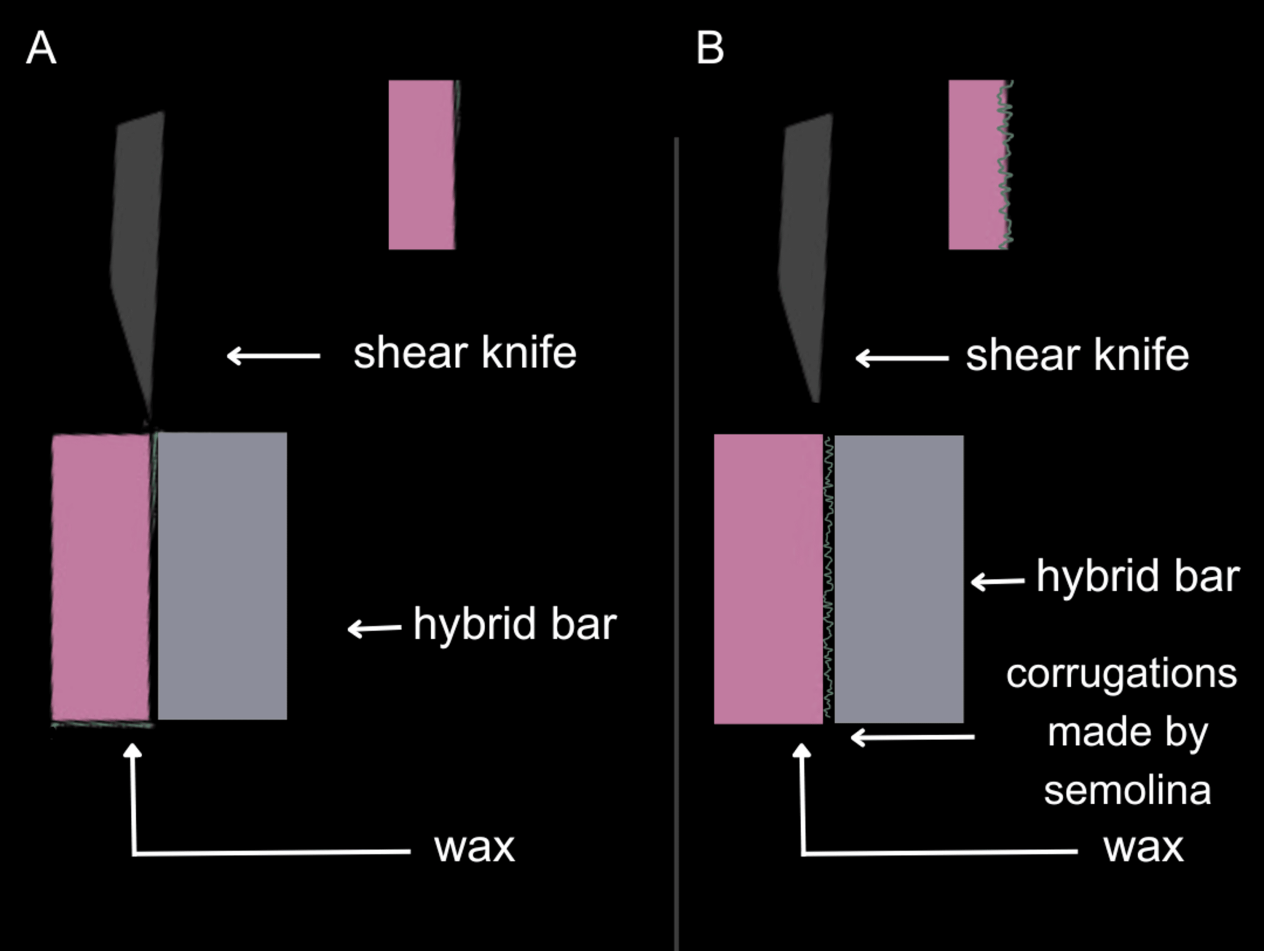
Testing apparatus

The fabricated samples were positioned in a universal testing machine (Instron Corp., Canton, USA; Model 3000) to gauge shear bond strength at a velocity of 0.5 mm/minute. Force was administered to the interface between the acrylic resin and alloy (Figure 5). The experimental setup included a double-mounted specimen and a 1 kN load cell, ensuring perpendicular alignment of the shear knife to the sample surface. The shearing process commenced 2 mm above the metal. The value of SBS, in MPa, was derived by dividing the failure load (N) by the total surface area of heat-polymerized acrylic resin (N/πr²).

Statistical Analysis

The SBS for all the groups was tabulated using Microsoft Excel (Microsoft Corp., Redmond, USA). To perform statistical analysis at a significance threshold of α=0.05, this data was imported into IBM SPSS Statistics for Windows version 26 (IBM Corp., Armonk, USA). The Shapiro-Wilk test was utilized to evaluate the data's normality. In order to compare all groups, the one-way analysis of variance (ANOVA) test was employed. To assess variations in groups, a post-hoc test was done.

## Results

The results of ANOVA revealed significant differences among various group comparisons (Table 2). For the 'Conventional Casting' group, the mean differences when compared to other groups were as follows: 'Conventional Casting with Opaquer' showed a mean difference of 0.42600 with a standard error of 0.18934, which was not statistically significant. In contrast, the mean difference with 'DMLS CoCr' was 2.600 with a standard error of 0.18934, indicating a significant difference. 'DMLS CoCr with Opaquer' had a mean difference of 1.877 and 'DMLS Ti' showed a significant mean difference of 3.368, both with a standard error of 0.18934. When compared to 'DMLS Ti with Opaquer', the mean difference was 0.91400. The mean difference with 'Semolina' was the highest at 7.13200, followed by 'Semolina with Opaquer' at 2.2400, all with the same standard error. Comparisons for the 'Conventional Casting with Opaquer' group showed significant differences with 'DMLS CoCr' (mean difference 3.02600), 'DMLS CoCr with Opaquer' (2.303), 'DMLS Ti' (3.794), and 'DMLS Ti with Opaquer' (1.3400). The differences were most pronounced with 'Semolina' (7.5580) and 'Semolina with Opaquer' (2.666).

**Table 2:** Mean and standard deviation among all groups * signifies p-value <0.05 DMLS: direct metal laser sintering; Ti: titanium; CoCr: cobalt-chromium

Group	Comparison Group	Mean Difference	Std. Error	Sig	95% Confidence Interval	
					Lower Bound	Upper Bound
Conventional Casting	Conventional Casting with Opaquer	0.42600	0.18934	0.335	1.651	1.0171
	DMLS CoCr	2.600	0.18934	0.000*	3.1911	2.0089
	DMLS CoCr with Opaquer	1.877	0.18934	0.000*	2.4681	1.2859
	DMLS Ti	3.368	0.18934	0.000*	3.9591	2.7769
	DMLS Ti with Opaquer	0.91400	0.18934	0.000*	1.5051	0.3229
	Semolina	7.13200	0.18934	0.000*	7.7231	6.5409
	Semolina with Opaquer	2.2400	0.18934	0.000*	2.8311	1.6489
Conventional Casting with Opaquer	DMLS CoCr	3.02600	0.18934	0.000*	3.6171	2.4349
	DMLS CoCr with Opaquer	2.303	0.18934	0.000*	2.8941	1.7119
	DMLS Ti	3.794	0.18934	0.000*	4.3851	3.2029
	DMLS Ti with Opaquer	1.3400	0.18934	0.000*	1.9311	0.7489
	Semolina	7.5580	0.18934	0.000*	8.1491	6.9669
	Semolina with Opaquer	2.666	0.18934	0.000*	3.2571	2.0749
DMLS CoCr	DMLS CoCr with Opaquer	0.723	0.18934	0.007*	0.1319	1.3141
	DMLS Ti	0.768	0.18934	0.003*	1.3591	0.1769
	DMLS Ti with Opaquer	1.68	0.18934	0.000*	1.0949	2.2771
	Semolina	4.532	0.18934	0.000*	5.1231	3.9409
	Semolina with Opaquer	0.36000	0.18934	0.555	0.2311	0.9511
DMLS CoCr with Opaquer	DMLS Ti	1.491	0.18934	0.000*	2.0821	0.8999
	DMLS Ti with Opaquer	0.9630	0.18934	0.000*	0.3719	1.5541
	Semolina	5.2550	0.18934	0.000*	5.8461	4.6639
	Semolina with Opaquer	0.363	0.18934	0.544	0.9541	0.2281
DMLS Ti	Semolina	3.764	0.18934	0.000*	4.3551	3.1729
	Semolina with Opaquer	1.128	0.18934	0.000*	0.5369	1.7191
DMLS Ti with Opaquer	Semolina	6.21800	0.18934	0.000*	6.8091	5.6269
	Semolina with Opaquer	1.32600	0.18934	0.000*	1.9171	0.7349

The 'DMLS CoCr' group compared to 'DMLS CoCr with Opaquer' showed a mean difference of 0.723, which was significant. When compared to 'DMLS Ti', the mean difference was 0.768. The difference increased with 'DMLS Ti with Opaquer' (1.68) and was highest with 'Semolina' (4.532). The comparison with 'Semolina with Opaquer' showed a smaller mean difference of 0.36000, which was not statistically significant. For the 'DMLS CoCr with Opaquer' group, comparisons with 'DMLS Ti' showed a significant mean difference of 1.491 while the difference with 'DMLS Ti with Opaquer' was 0.9630. The differences between 'Semolina' and 'Semolina with Opaquer' were substantial, at 5.2550 and 0.363, respectively. The 'DMLS Ti' group showed significant mean differences when compared to 'Semolina' (3.764) and 'Semolina with Opaquer' (1.128). Finally, the 'DMLS Ti with Opaquer' group showed significant differences with 'Semolina' (6.21800) and 'Semolina with Opaquer' (1.32600), indicating that the presence of opaquer had a considerable impact on the outcomes across different material groups. 'Conventional Casting with Opaquer' significantly outperformed all groups, especially 'Semolina', which showed the highest mean difference (7.558). Similar trends were seen with 'DMLS CoCr' and 'DMLS Ti', where 'Semolina' consistently demonstrated superior bond strength.

**Figure 6:**
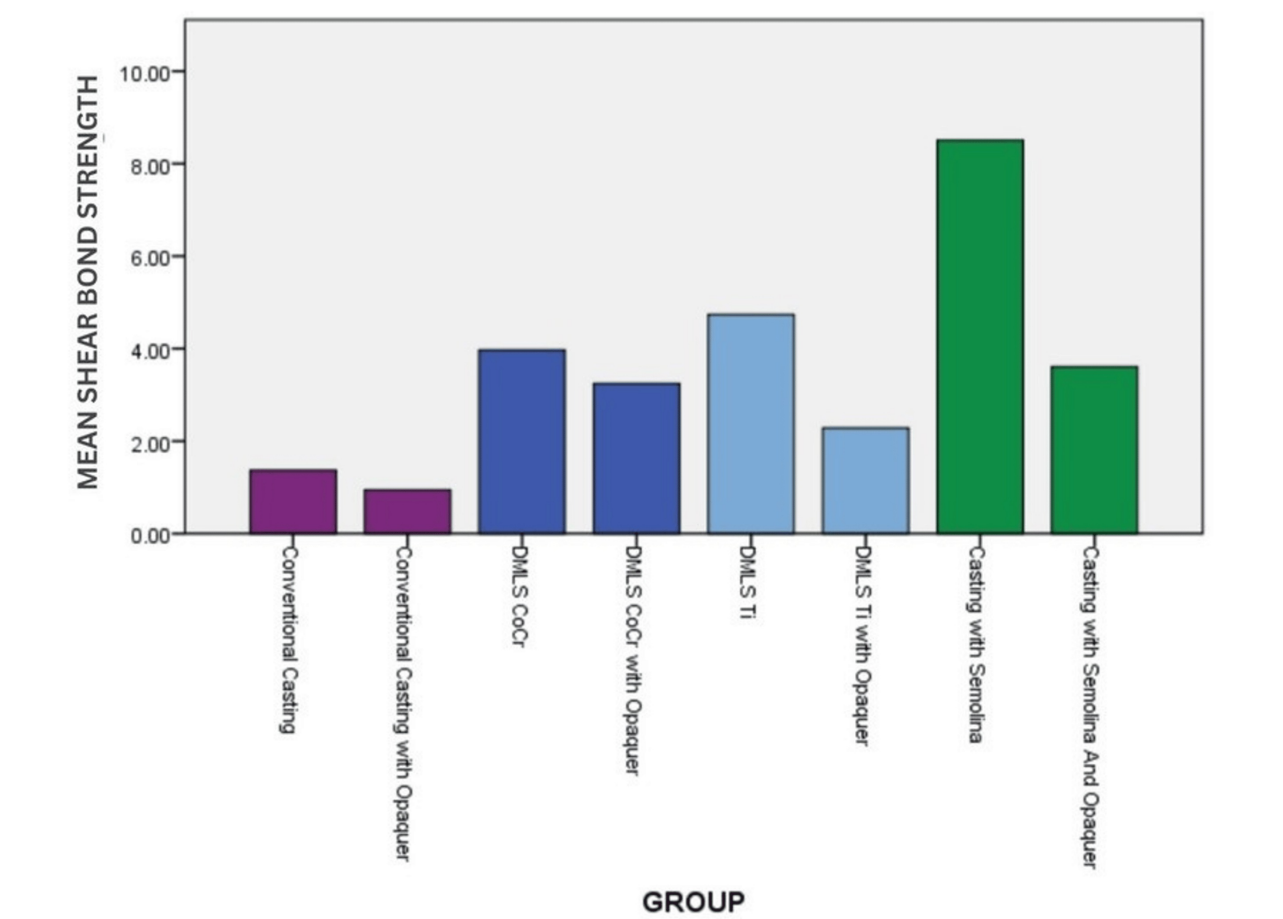
Graph demonstrating shear bond strength values across all groups DMLS: direct metal laser sintering; Ti: titanium; CoCr: cobalt-chromium

Overall, 'Semolina' produced the highest bond strengths across all conditions. The use of opaquer demonstrated reduced values in all the groups. These results underscore the importance of material selection and treatment in enhancing bond strength (Figure 6).

## Discussion

The main objective of this research was to assess the efficiency of semolina in increasing the shear bond strength between metal alloys and denture base resins. It provides a clear picture of the bond strength varying among materials and holds great clinical significance owing to the large number of failures observed at the acrylic-metal interface. The maximum shear bond strength was seen in the 'Semolina' group, and therefore, the null hypothesis was rejected. The complete burnout of semolina allowed for investigation free of impurities. Among the specimens with and without the use of opaquer, the samples with opaquer showed significantly lower values. The opaquer is often used to avoid the hue of metal seen through acrylic. This could be considered a common reason for failure wherein aesthetics are given priority over the life of restoration. 

A study by Johansson et al. reported a fracture of acrylic to substructure in 22 patients included in their study over a mean of 5.2 years [7]. Many authors have reported results based on the use of primers to improve bond strength. In a study by Matsuda et al., the use of primer after air abrasion consisting of a hydrophobic phosphate monomer significantly increased the SBS of acrylic to Ti and CoCr alloy [8]. In a study by Kawaguchi et al., CoCr samples were categorized into five categories: (a) treated with air abrasion using alumina, (b) coated with the Rocatec tribochemical silica system, (c) Epricord opaque primer application after air abrasion, (d) application of Super-Bond C&B liquid after air abrasion, and (e) alloy primer application after air abrasion. Each specimen underwent up to 10,000 thermocycles after acrylization. After thermocycling, the alumina group had a significantly higher shear bond strength than the other groups [9].

Another study found that air abrasion greatly increased the SBS for samples fabricated by milling and casting (p<0.001), with SBS being higher for larger-sized aluminum oxide particles. Milled Ti exhibited higher SBS values than cast Ti when untreated with alumina particles as well as when treated with smaller particles (50 µm) [10]. Primers enhanced the shear bond strengths of denture base resin to all metals (p<0.05), with no significant differences observed among the metals. Post-thermocycling, there was a notable reduction in bond strengths across all groups. MetaBond exhibited the least durability, with bond strength decreases ranging from 22.8% to 35.5% among the primers [11]. 

When compared to bench curing, pressure-pot curing usually showed higher SBS. The majority of the specimens that were primed showed cohesive bond failures within the primer or acrylic resin, whereas the non-primed specimens typically had adhesive bond failures at the interface between the resin and metal [12]. Silicoating specimens prior to acrylic specimens did not show statistical significance on shear bond strength values, whereas the Kevloc system provided better results [13]. The SBS of the acrylic resin to the base metal alloy was significantly higher than the SBS to the noble and Ti alloys [14]. Alloy type influences the shear bond strength of the denture base resin to silicoated alloys, but no difference in bond strength was found between gold-palladium (Au-Pd), gold-silver-palladium-copper (Au-Ag-Pd-Cu), high-palladium (high-Pd), and nickel-chromium-beryllium (Ni-Cr-Be) alloys [15].

Spark erosion produced substantially higher bond strength values on Ti compared to Rocatec, with spark erosion yielding the highest average bond strengths across all metals. These findings, corroborated by scanning electron microscopy (SEM) analysis, demonstrated that sandblasting is unnecessary for achieving strong bond strength with spark erosion [16]. Among different brands of resins, Trutone resin showed high SBS in non-primed groups in comparison to the other resins. When used along with Dentsply primer, the Rocatec-treated Lucitone 199 resin had the highest bond strength [17].

Acknowledging the limitations of this study, it is important to note that the forces acting on the prosthesis intraorally will differ from those in an in vitro evaluation. Intraoral forces will be both compressive and torsional, which may lead to different stress patterns at the metal-acrylic junction. Over time, these stresses could potentially reduce the shear bond strength of the prosthesis as it undergoes aging. Commercially available semolina is sold in various grain sizes, which can affect the surface corrugations produced on the alloy surface. Therefore, standardizing the grade and type of semolina used may be challenging.

## Conclusions

Hybrid techniques combining digital and conventional workflow in dentistry in developing nations require economic scaling of our dental practices and laboratories. The fracture at the metal-acrylic junction is a relevant issue in hybrid prostheses that is supported by multiple implants and is a problem that requires to be solved. Over time, numerous primers have been developed to address this problem, but they often fail after prolonged use. Additionally, attempts to improve mechanical retention through digital attachment designs have not been successful. These designs are difficult to mill and tend to break easily during sandblasting. In this study, the primary goal was to evaluate a method to improve the bond strength at the metal-acrylic interface. The novel method to increase shear bond strength between acrylic and metal was the use of semolina to increase surface corrugations of the alloy surface. This approach is both economical and reliable. Semolina is widely available and inexpensive, making it accessible for use globally. During the dewaxing process, semolina burns out completely without leaving any residue, making it an excellent material for enhancing mechanical bonding between acrylic and metal. The application of semolina in the bonding process is straightforward, which could simplify procedures and reduce the risk of errors during the manufacturing of prostheses. 
